# The Phosphatase PTPL1 Is Required for PTEN-Mediated Regulation of Apical Membrane Size

**DOI:** 10.1128/MCB.00102-18

**Published:** 2018-05-29

**Authors:** Lucas J. M. Bruurs, Mirjam C. van der Net, Susan Zwakenberg, Axel K. M. Rosendahl Huber, Anneke Post, Fried J. Zwartkruis, Johannes L. Bos

**Affiliations:** a Oncode Institute, Center for Molecular Medicine, University Medical Center Utrecht, Utrecht University, Utrecht, The Netherlands

**Keywords:** cell polarity, PTEN, PTPL1

## Abstract

PTEN is a tumor suppressor that is frequently lost in epithelial malignancies. A part of the tumor-suppressive properties of PTEN is attributed to its function in cell polarization and consequently its role in maintaining epithelial tissue integrity. However, surprisingly little is known about the function and regulation of PTEN during epithelial cell polarization. We used clustered regularly interspaced short palindromic repeat (CRISPR)/Cas9-mediated gene disruption to delete PTEN in intestinal epithelial Ls174T:W4 cells, which upon differentiation form a microvillus-covered apical membrane (brush border) on a part of the cell cortex, independent of cell-cell junctions. We show that loss of PTEN results in the formation of a larger brush border that, in a fraction of the cells, even spans the entire plasma membrane, revealing that PTEN functions in the regulation of apical membrane size. Depletion of the phosphatase PTPL1 resulted in a similar defect. PTPL1 interacts with PTEN, and this interaction is necessary for apical membrane enrichment of PTEN. Importantly, phosphatase activity of PTPL1 is not required, indicating that PTPL1 functions as an anchor protein in this process. Our work thus demonstrates a novel function for PTEN during cell polarization in controlling apical membrane size and identifies PTPL1 as a critical apical membrane anchor for PTEN in this process.

## INTRODUCTION

Control over cell polarization contributes to the maintenance of epithelial tissue integrity and thereby provides a barrier in tumorigenesis (1–3). The tumor suppressor PTEN is frequently lost in epithelial malignancies, and loss of PTEN often coincides with loss of epithelial tissue integrity (4, 5). PTEN is a lipid phosphatase that is implicated primarily in the conversion of phosphatidylinositol-3,4,5 triphosphate [PI(3,4,5)P_3_] to phosphatidylinositol-4,5 bisphosphate [PI(4,5)P_2_], thereby counteracting the activity of phosphatidylinositol 3-kinase (PI3K) in the Akt/protein kinase B (PKB) signaling pathway (6, 7).

In addition, PTEN has a role in the regulation of cell polarization via which it contributes to epithelial tissue integrity (8–14). However, how PTEN controls cell polarity and how it is regulated have not been clearly established. For instance, Martin-Belmonte et al. reported that PTEN is required for the proper formation of an apical membrane and is necessary for normal lumen formation in MDCK cyst cultures (14). Similar results were subsequently reported using three-dimensional (3D) mammary gland cultures and CaCO2 cysts (10, 15). However, recent findings in (inducible) PTEN knockout mice demonstrate that although lumen morphology is altered, apical membrane formation is not impaired in epithelial cells that have lost PTEN (8, 9).

PTEN controls various interconnected aspects of epithelial cell polarization, including apical domain specification and junction formation, and this could account for the different phenotypes observed in various model systems (13, 14, 16). We studied the role of PTEN in cell polarization in the Ls174T:W4 colon carcinoma cell line (17). These cells polarize and form a microvillus-covered apical membrane (brush border) in the absence of cell-cell junctions after forced activation of LKB1 by doxycycline-induced expression of its coactivator STRADα (17). Because of this feature of Ls174T:W4 cells, it is possible to uncouple cell-intrinsic PTEN signaling from junction-dependent PTEN signaling.

We show that loss of PTEN results in an enlarged apical membrane, implying a critical role for PTEN in the regulation of apical membrane size. Furthermore, we identified PTPL1, a protein tyrosine phosphatase and putative tumor suppressor protein (18), as a PTEN binding partner required in this process.

## RESULTS

**FIG 1:**
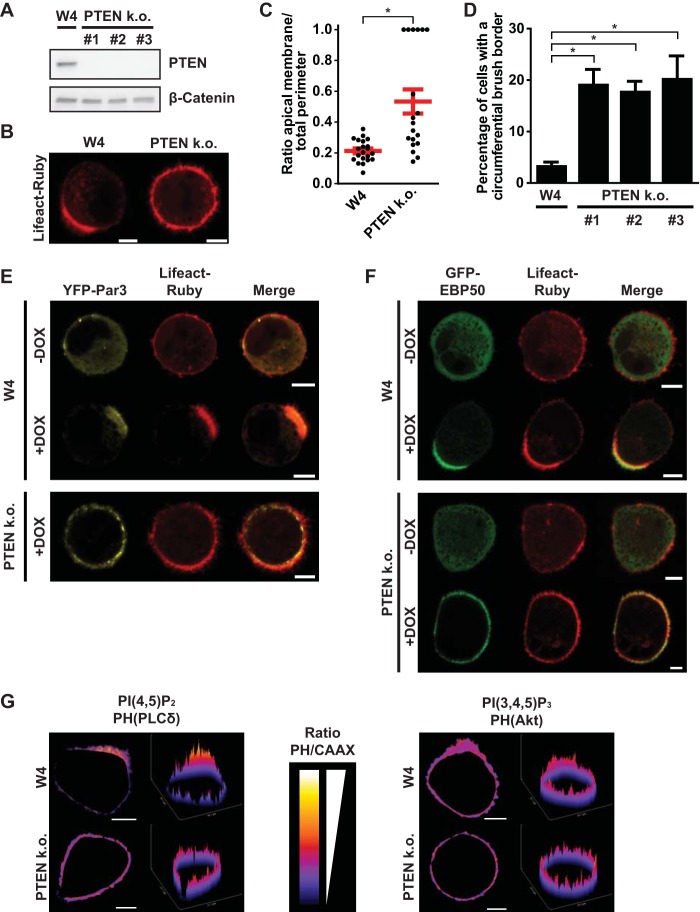
PTEN knockout (k.o.) W4 cells cannot restrict apical membrane formation. (A) Western blot of W4 cell and PTEN k.o. cell lysates probed for PTEN and β-catenin. (B) Localization of the actin marker Lifeact-Ruby in polarized W4 cells and PTEN k.o. cells. Scale bars, 5 μm. (C) Quantification of apical membrane size in doxycycline-stimulated W4 cells and PTEN k.o. cells. Red bars represent the average. Error bars represent the standard error of the mean (SEM) (*n* > 19). *, *P* < 0.05 using independent sample *t* tests. (D) Quantification of the fraction of cells that form an apical plasma membrane that covers the entire cell perimeter in W4 cells and PTEN knockout cells based on GFP-EBP50 and Lifeact-Ruby localization. Error bars represent SEM in three experiments (*n* > 100 cells per experiment). *, *P* < 0.05 using independent sample *t* tests. (E) Localization of the apical determinant YFP-Par3 and Lifeact-Ruby in unpolarized (−DOX) and polarized (+DOX) W4 cells and DOX-treated PTEN k.o. cells. Scale bars, 5 μm. DOX, doxycycline. (F) Localization of the brush border marker GFP-EBP50 and Lifeact-Ruby in unpolarized (−DOX) and polarized (+DOX) W4 cells and PTEN k.o. cells. Scale bars, 5 μm. (G) Ratio images and surface plots of PH-PLCδ-RFP/GFP-Kras(CAAX) [reflecting PI(4,5)P_2_ distribution] (left) and PH-Akt-GFP/RFP-Kras(CAAX) [reflecting PI(3,4,5)P_3_ distribution] (right) in polarized W4 and PTEN k.o. cells. Scale bars, 5 μm.

To study the function of PTEN during epithelial cell polarization, we established PTEN knockout Ls174T:W4 cells using clustered regularly interspaced short palindromic repeat (CRISPR)/Cas9-mediated gene disruption (Fig. 1A). In general, doxycycline-induced polarization of Ls174T:W4 cells results in the formation of a basolateral domain and a microvillus-covered apical domain (17). In contrast, doxycycline-stimulated PTEN knockout cells often formed a brush border that was enlarged compared to that of normal Ls174T:W4 cells (Fig. 1B and C). Strikingly, in a fraction of PTEN knockout cells, microvilli covered the complete cell perimeter, suggesting that these cells form only an apical membrane (Fig. 1B, C, and D).

To demonstrate that the microvilli in PTEN knockout cells indeed represent a bona fide apical brush border, we assessed the distributions of the apical membrane determinant yellow fluorescent protein (YFP)-Par3 and the brush border marker green fluorescent protein (GFP)-EBP50 in these cells (16, 19). In unstimulated (i.e., unpolarized) Ls174T:W4 cells, YFP-Par3 is mostly cytosolic, but upon doxycycline-induced polarization, YFP-Par3 localization is restricted to the apical membrane. In contrast, in doxycycline-stimulated PTEN knockout cells, YFP-Par3 covers the entire cell cortex, indicating the formation of an apical membrane that spans the entire cell perimeter (Fig. 1E). Similarly, whereas the brush border marker GFP-EBP50 distributes uniformly in the cytosol of unpolarized cells, it is located exclusively in the brush border in polarized Ls174T:W4 cells (Fig. 1F). In unstimulated PTEN knockout cells GFP-EBP50 also is mostly cytosolic, but upon doxycycline stimulation, GFP-EBP50 is recruited to the entire plasma membrane, demonstrating that a fraction of PTEN knockout cells form an apical brush border that covers the entire surface of the cell (Fig. 1F).

In polarized epithelial cells, PTEN establishes the asymmetric distribution of phosphoinositide membrane lipids and thereby contributes to apical and basolateral domain identity (14, 20, 21). To test whether PTEN is required for PI(4,5)P_2_ and PI(3,4,5)P_3_ gradients in polarized Ls174T:W4 cells, we assessed the distribution of the pleckstrin homology (PH) domains of phospholipase C-δ (PLCδ) and Akt to determine the localization of PI(4,5)P_2_ and PI(3,4,5)P_3_, respectively. However, to exclude geometrical bias of the membrane-rich brush border, we normalized the intensity of the fluorescent PH domain to the intensity of a membrane marker [Kras(CAAX)].

In agreement with previous findings, we found that PI(4,5)P_2_ is enriched at the apical membrane compared to the basolateral domain (Fig. 1G) (14). This gradient is lost in PTEN knockout cells, which form an apical membrane that spans that entire cell perimeter, suggesting that PTEN may regulate apical membrane size by establishing a PI(4,5)P_2_ gradient (Fig. 1G). In contrast, no apparent gradient in PI(3,4,5)P_3_ distribution was observed in either normal Ls174T:W4 cells or PTEN knockout cells (Fig. 1G).

**FIG 2:**
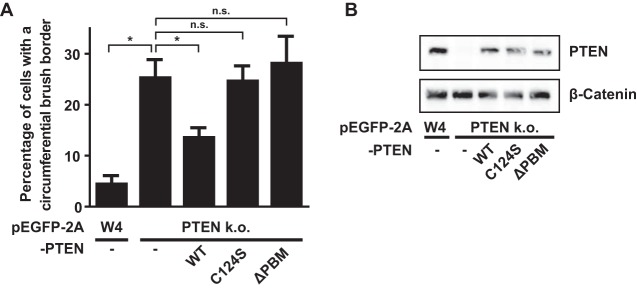
The PDZ binding motif of PTEN is required for apical membrane clustering. (A) Quantification of the fraction of cells that form a brush border that covers the entire cell perimeter in W4 cells and PTEN k.o. cells expressing PTEN wt, C124S, or ΔPBM based on mCherry-EBP50 localization. Error bars represent SEM in three experiments (*n* > 100 cells per experiment). *, *P* < 0.05 using independent sample *t* tests. n.s., not significant (*P* > 0.05). (B) Western blot of W4 cells and PTEN knockout cells expressing PTEN wt, C124S, or ΔPBM probed for PTEN and β-catenin.

Next, we tested whether the phosphatase activity and the C-terminal PDZ binding motif (PBM) of PTEN are important for PTEN′s ability to control apical membrane size. For this, we expressed either wild-type (wt) PTEN, catalytically inactive C124S, or PTENΔPBM in PTEN knockout cells and quantified the fraction of cells that formed an apical membrane that covered the complete cell perimeter. We found that expression of wild-type PTEN resulted in a partial rescue of the PTEN knockout phenotype, which could suggest that PTEN dosage is important in the regulation of apical membrane size (Fig. 2A). Nonetheless, expression of PTEN(C124S) or PTENΔPBM did not lead to a similar decrease in the fraction of cells with a circumferential brush border (Fig. 2). Therefore, we conclude that the phosphatase activity and regulation via the C-terminal PDZ binding motif are required for PTEN-dependent regulation of apical membrane size.

**FIG 3:**
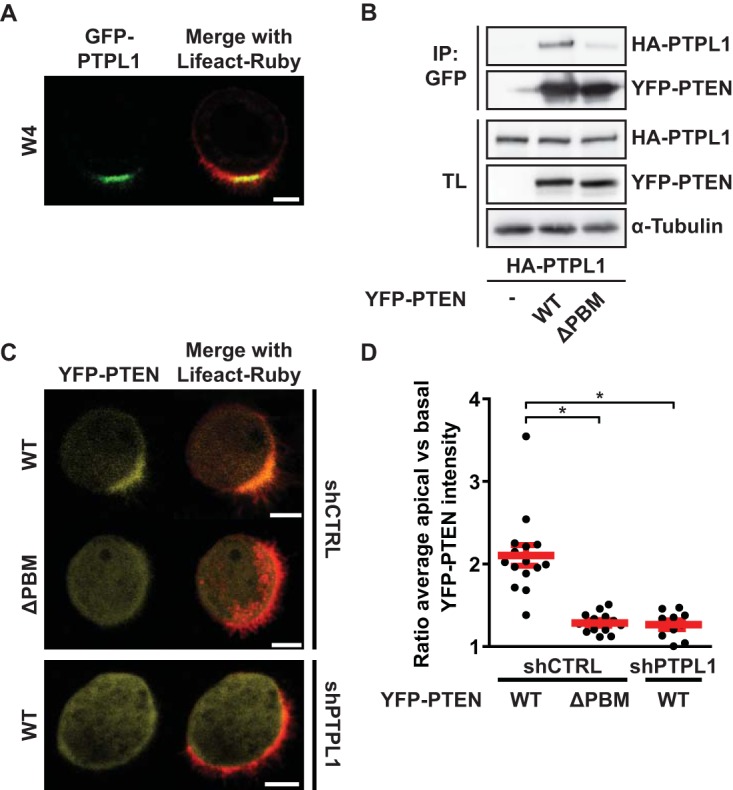
PTPL1 binds PTEN and ensures apical enrichment of PTEN. (A) Localization of GFP-PTPL1 and Lifeact-Ruby in polarized W4 cells. Scale bar, 5 μm. (B) Coimmunoprecipitation (IP) of HA-PTPL1 with YFP-PTEN wt and ΔPBM in HEK293T cells. TL, total lysate. (C) Localization of YFP-PTEN wt and ΔPBM in W4:shCTRL cells and of YFP-PTEN wt in PTPL1-depleted cells in combination with Lifeact-Ruby. Scale bars, 5 μm. (D) Quantification of apical enrichment of YFP-PTEN wt and ΔPBM in control or PTPL1-depleted W4 cells. Red bars represent the average, and error bars represent the SEM (*n* > 10). *, *P* < 0.05 using independent sample *t* tests.

Having demonstrated that the PDZ binding motif of PTEN is crucial for PTEN′s function in limiting apical membrane formation to a confined domain, we next focused on possible interaction partners that may be important for this function of PTEN. PTPL1 is a large multidomain protein tyrosine phosphatase that localizes to the apical membrane of polarized epithelial cells and is able to bind PTEN *in vitro* via its second PDZ domain (Fig. 3A) (22, 23). Coimmunoprecipitation of PTPL1 with PTEN in HEK293T cells revealed that the full-length proteins interact, and this binding was largely reduced when the PDZ binding motif of PTEN was deleted (Fig. 3B).

To assess the consequence of the interaction between PTEN and PTPL1, we determined the localization of YFP-PTEN and YFP-PTENΔPBM in polarized Ls174T:W4 cells. We found that whereas YFP-PTEN is concentrated at the apical membrane, YFP-PTENΔPBM does not show a similar enrichment (Fig. 3C). Furthermore, in cells stably depleted of PTPL1, localization of YFP-PTEN mimics the diffuse localization of YFP-PTENΔPBM (Fig. 3C). We quantified this by determining the ratio of average apical and basal YFP-PTEN pixel intensities and found that PTEN requires its PBM and the presence of PTPL1 to become apically enriched, indicating that binding with PTPL1 is important for apical PTEN localization (Fig. 3D).

**FIG 4:**
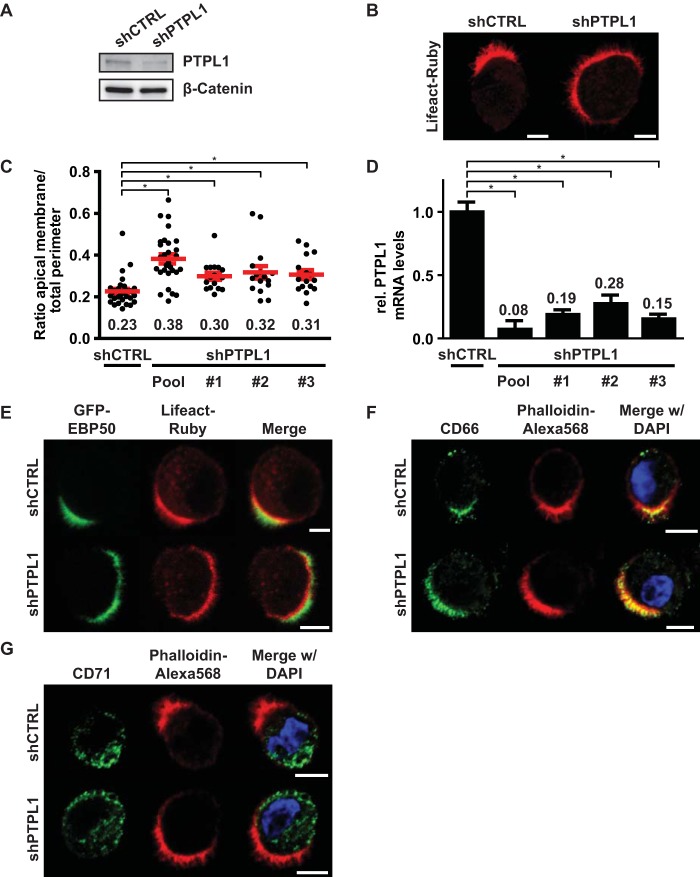
PTPL1 is required for apical membrane clustering. (A) Western blot of W4:shCTRL or W4:shPTPL1 lysates probed for PTPL1 and β-catenin. (B) Localization of Lifeact-Ruby in W4:shCTRL or PTPL1-depleted W4 cells. Scale bars, 5 μm. (C) Quantification of apical membrane size in W4:shCTRL and W4:shPTPL1 cells. Red bars represent the average. Red error bars represent the SEM (*n* > 17). *, *P* < 0.05 using independent sample *t* tests. (D) Relative PTPL1 mRNA expression levels as determined by qPCR. Values in the graph represent the average. Error bars represent the SEM (*n* = 6; 2 biological and 3 technical replicates). *, *P* < 0.05 using independent sample *t* tests. (E, F, and G) Localization of the brush border marker GFP-EBP50 (E), apical membrane marker CD66 (F), and basolateral domain marker CD71 (G) in polarized W4:shCTRL and W4:shPTPL1 cells in combination with the actin marker Lifeact-Ruby or phalloidin-Alexa Fluor 568 and DAPI. Scale bars, 5 μm.

Next, we tested whether PTPL1 has a function in controlling apical membrane size similar to that of PTEN. Although the effect was less severe than that in PTEN knockout cells, PTPL1-depleted cells also formed enlarged apical membranes, indicating that both proteins are required to control apical membrane size (Fig. 4A to D). Similar to PTEN knockout cells, the microvilli on PTPL1-depleted cells were positive for GFP-EBP50, demonstrating that they represent a genuine apical brush border (Fig. 4E). In addition, segregation of apical and basolateral proteins still occurred in PTPL1-depleted cells as judged by the distribution of apical membrane (CD66) and basolateral domain (CD71) markers (Fig. 4F and G). These experiments therefore demonstrate that PTPL1 is required for the clustered formation of an apical membrane.

**FIG 5:**
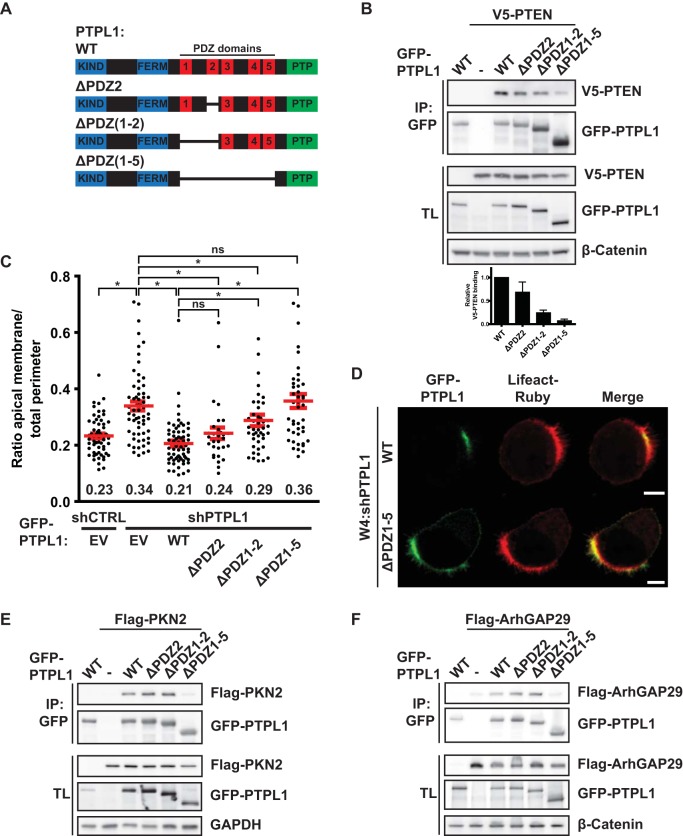
PTPL1 binding is required for PTEN-dependent apical membrane clustering. (A) Schematic representation of PTPL1 mutants. KIND, kinase noncatalytic C-lobe domain; FERM, 4.1, ezrin, radixin, moesin domain; PTP, protein tyrosine phosphatase domain. (B) Coimmunoprecipitation of V5-PTEN with GFP-PTPL1 wt and PDZ deletion mutants in HEK293T cells. Bottom, quantification of relative V5-PTEN binding to GFP-PTPL1. Error bars represent the SEM (*n* = 4). (C) Quantification of apical membrane size in W4:shCTRL and W4:shPTPL1 cells expressing EV, GFP-PTPL1 wt, or PDZ deletion mutants. Red bars represent the average. Error bars represent the SEM (*n* > 30 in at least three experiments). *, *P* < 0.05 using independent sample *t* tests. ns, not significant (*P* > 0.05). EV, empty vector. (D) Localization of GFP-PTPL1, GFP-PTPL1(ΔPDZ1-5), and Lifeact-Ruby in PTPL1-depleted W4 cells. Scale bars, 5 μm. (E) Coimmunoprecipitation of Flag-PKN2 with GFP-PTPL1 wt and PDZ deletion mutants in HEK293T cells. (F) Coimmunoprecipitation of Flag-ArhGAP29 with GFP-PTPL1 wt and PDZ deletion mutants in HEK293T cells.

In order to test the hypothesis that PTPL1 controls apical domain size by binding PTEN, we generated PTPL1 mutants in which the PDZ domains responsible for PTEN binding were deleted (Fig. 5A). Although the second PDZ domain of PTPL1 is the only PDZ domain that can bind PTEN *in vitro* (23), deletion of PDZ2 only marginally reduced PTEN binding (Fig. 5B). Therefore, we additionally deleted PDZ1 and the region between PDZ1 and PDZ2 (“interregion”), which modulates PTEN binding *in vitro* (23), resulting in a further reduction of PTEN binding. Only deletion of all five PDZ domains resulted in a near-complete loss of PTEN binding, indicative of redundancy between PDZ domains for PTEN binding (Fig. 5B).

Next, we tested whether the PTEN-binding-defective PTPL1 mutants could restore normal apical membrane size in Ls174T:W4 cells in which endogenous PTPL1 was silenced. We found that the degree of rescue correlated with the ability to bind PTEN: whereas expression PTPL1(ΔPDZ2) restored normal brush border size to the same extent as wild-type PTPL1, PTPL1(ΔPDZ1-5) was unable to rescue PTPL1 depletion. Expression of PTPL1(ΔPDZ1-2) resulted in a partial rescue of apical membrane size (Fig. 5C). As was previously reported, GFP-PTPL1(ΔPDZ1-5) is localized primarily at the apical plasma membrane, indicating that deletion of the PDZ domains does not greatly affect PTPL1 localization (Fig. 5D) (22). Furthermore, both PTPL1(ΔPDZ2) and PTPL1(ΔPDZ1-2) retain the ability to bind PKN2 and ArhGAP29, which bind PTPL1 via the third and fourth PDZ domains, respectively, indicating that these deletions have limited impact on protein structure (Fig. 5E and F) (24, 25). These findings therefore further support the hypothesis that PTEN binding to PTPL1 is required to control apical membrane size.

**FIG 6:**
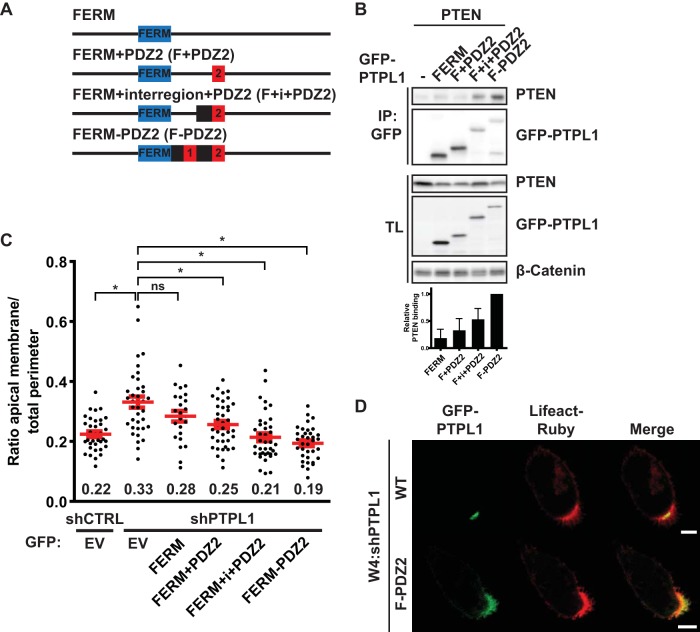
A minimal version of PTPL1, composed of the FERM domain and the PTEN binding region, is able to rescue apical membrane clustering in PTPL1-depleted cells. (A) Schematic representation of minimal PTPL1 mutants. FERM, 4.1, ezrin, radixin, moesin domain. (B) Coimmunoprecipitation of PTEN with minimal PTPL1 versions in HEK293T cells. Bottom, quantification of relative PTEN binding normalized to GFP-FERM-PDZ2. Error bars represent the SEM (*n* = 4). (C) Quantification of apical membrane size in W4:shCTRL and W4:shPTPL1 cells expressing EV or GFP-PTPL1 mutants. Red bars represent the average. Error bars represent the SEM (*n* > 24 in at least three experiments). *, *P* < 0.05 using independent sample *t* tests. ns, not significant (*P* > 0.05). (D) Localization of GFP-PTPL1, GFP-FERM-PDZ2, and Lifeact-Ruby in PTPL1-depleted W4 cells. Scale bars, 5 μm.

To further test whether the ability to bind PTEN is sufficient for PTPL1 function in controlling apical domain size, we generated minimal versions of PTPL1 composed of its localization signal, the FERM domain, and the PDZ domains responsible for PTEN binding (Fig. 6A). In agreement with the results with the PDZ deletion mutants, PTEN bound poorly to PDZ2 but binding increased when the interregion and PDZ1 were added (Fig. 6B). Expression of these minimal PTPL1 constructs in PTPL1-depleted Ls174T:W4 cells resulted in normalization of apical membrane size in a manner that correlated with the ability to bind PTEN (Fig. 6C). In support of a model in which PTPL1 functions as an apical scaffold for PTEN, localization of the GFP-FERM-PDZ2 construct, which fully rescues PTPL1 depletion, was almost exclusively apical (Fig. 6D). Therefore, these experiments indicate that PTPL1 controls apical membrane size by binding PTEN at the apical plasma membrane.

**FIG 7:**
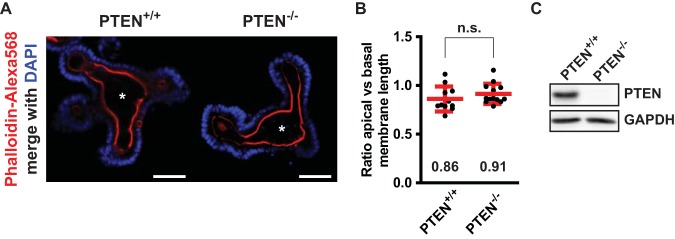
Deletion of PTEN in mouse small intestinal organoids does not result in severe morphological defects. (A) Uninduced (PTEN^+/+^) and induced (PTEN^−/−^) mouse small intestinal organoids stained for actin (phalloidin-Alexa Fluor 568) and DNA (DAPI). Scale bars, 50 μm. Asterisks indicate the position of the central lumen. (B) Ratio of apical versus basal membrane length of enterocytes that line the central lumen. Red bars represent the average. Error bars represent the standard deviation (SD) (*n* > 11 organoids in at least two experiments). n.s., not significant using independent sample *t* tests (*P* > 0.05). (C) Western blot of uninduced and induced organoid lysates probed for PTEN and GAPDH.

Since Ls174T:W4 cells polarize in the absence of cell-cell junctions, we tested to what extent PTEN is required to control the apical membrane in mouse small intestinal organoids, which form a fully polarized intestinal epithelial monolayer with normal cell junctions (26). We previously demonstrated that loss of control over apical membrane size in enterocytes results in the formation of an aberrantly shaped central lumen in mouse intestinal organoids (27). However, we could not find similar morphological abnormalities in organoids in which PTEN was deleted, indicating that in the context of an intact monolayer, additional regulation of apical membrane size is present, which can compensate for the loss of PTEN (Fig. 7).

## DISCUSSION

Here we show that PTEN controls apical membrane size in polarized Ls174T:W4 cells. For this, PTEN, by means of its PDZ binding motif, binds to the PDZ domains of PTPL1, which in turn ensures apical enrichment of PTEN. Abrogating the interaction between PTEN and PTPL1 results in diffuse PTEN localization and enlargement of the apical membrane. These data therefore support a model where PTPL1, by binding and localizing PTEN to the apical membrane, enables PTEN-dependent restriction of apical membrane size. This work thus identifies a novel function for PTEN during cell polarization in controlling apical membrane size and identifies PTPL1 as a critical PTEN binding partner in this process. It should be noted that our model system is unique in that polarization occurs in the absence of cell-cell junctions, which may reveal a function of PTEN which is masked in other model systems. Indeed, we did not observe obvious morphological defects in PTEN-deleted small intestinal organoids, suggesting that junction-dependent signaling can compensate for PTEN loss in the regulation of apical membrane size. Importantly, however, deletion of PTEN from the mouse retina and neural plate also resulted in enlarged cell surfaces, indicating that PTEN does regulate apical membrane size *in vivo* as well (9, 28).

How PTEN regulates apical membrane size remains elusive, but we find that PTEN is required to enforce the PI(4,5)P_2_ gradient that exists between the apical and basolateral membranes. PI(4,5)P_2_ is an early apical landmark and functions as a docking site for various apical signaling proteins (14, 29). Demonstrating this pioneering function of PI(4,5)P_2_ is the finding that exogenous addition of PI(4,5)P_2_ is sufficient to convert a basolateral membrane to an apical membrane (14). Therefore, we speculate that PTEN regulates apical membrane size by establishing a PI(4,5)P_2_ gradient.

Whereas others have reported a similar role for PI(3,4,5)P_3_ in governing basolateral membrane formation, we could not find an apparent PI(3,4,5)P_3_ gradient in polarized Ls174T:W4 cells (20, 30). The origin of this difference remains unclear, but it does suggest that PI(4,5)P_2_ is the dominant signaling lipid during Ls174T:W4 cell polarization.

Curiously, two cell populations are apparent in our PTEN knockout cell lines. The majority of cells form a partially clustered apical membrane, whereas a fraction of cells form an apical membrane that spans the complete cell perimeter. This bimodal response to PTEN deletion is apparent in all monoclonal knockout cell lines that we generated and is therefore probably not due to genetic variations in our cell cultures. Instead we speculate that the two populations originate from a threshold in the clustering process: we rarely observe cells that have a partially clustered apical membrane that covers more than 60% of the cell perimeter, suggesting that below this degree of apical membrane clustering, a cell will form a circumferential brush border.

The PDZ binding motif of PTEN engages in a variety of interactions with PDZ domain-containing proteins, including important regulators of cell polarization such as Par3, Dlg1, and MAGI-2 (31–33). We now show that PTPL1 is a functionally relevant interaction partner of PTEN in the regulation of apical membrane size. Importantly, only the FERM domain and the PDZ domains of PTPL1 are required, showing that in this process, PTPL1 functions to tether PTEN to the apical membrane and not as a tyrosine phosphatase. Whether other PTEN interactors are also involved in this process needs further investigation.

Both PTPL1 and PTEN function as tumor suppressors in colorectal cancer, raising the question to what extent their interaction is important for tumorigenesis (4, 18). Interestingly, two findings suggest that PTEN regulation via its PBM particularly contributes to tumorigenesis. First, PTEN^ΔPBM/ΔPBM^ mice develop spontaneous tumors and show higher tumor incidence when combined with various tumor mouse models (32). Second, although rare, oncogenic PTEN mutations that compromise the PBM have been reported, and these cannot restore the normal lumen morphology of PTEN-depleted 3D mammary gland cultures (11). Therefore, the interaction between PTPL1 and PTEN may be of relevance for cancer progression by controlling epithelial tissue architecture.

## MATERIALS AND METHODS

### Cell and organoid culture.

Ls174T:W4 cells were cultured in RPMI 1640 (Sigma) medium supplemented with 10% fetal bovine serum (FBS) (Sigma) and antibiotics. To induce polarization, cells were cultured for at least 16 h in the presence of 1 μg/ml doxycycline (Sigma). For transient expression of DNA constructs, cells were transfected using XtremeGene9 (Roche) according to the manufacturer's guidelines.

Small intestinal organoids from Pten^fl/fl^, villin CreER mice were provided by Owen Sansom. Organoids were suspended in Matrigel (BD) and cultured in advanced Dulbecco modified Eagle medium (DMEM)–F-12 medium (Life Technologies) supplemented with B27 supplement (Life Technologies), mouse epidermal growth factor (mEGF) (Life Technologies), R-Spondin-conditioned medium, Noggin-conditioned medium, and *N*-acetylcysteine. Every 5 days, organoid crypts were passaged by mechanical dissociation. PTEN deletion was induced by treating the organoids with 1 μM tamoxifen for 24 h.

### Plasmids.

pEGFP-EBP50 was generated by introducing an EBP50 PCR fragment in pEGFP-C2 using In-Fusion (Clontech). Similarly, PTPL1 constructs were generated by introducing a PTPL1 PCR product in pEGFP using In-Fusion. PTPL1 mutants were generated by performing the In-Fusion reaction with two PCR fragments, one fragment encoding the upstream sequence and one encoding the sequence downstream of the PDZ domain(s) to be deleted. For rescue experiments, wtpEGFP-2A-PTEN and the ΔPBM mutant were generated by generating a PCR fragment of PTEN in which PTEN is preceded by the sequence encoding the self-cleaving 2A peptide. This fragment was introduced in pEGFP-C2 using In-Fusion. For localization and coimmunoprecipitation experiments, YFP-PTEN, Flag-PKN2, and Flag-ArhGAP29 were generated using Gateway cloning (Invitrogen). PH-PLCδ-red fluorescent protein (RFP) and PH-Akt-GFP were provided by Tamas Balla, and Lifeact-Ruby was provided by Roland Wedlich-Söldner.

### Antibodies.

The following antibodies were used for immunofluorescence: mouse anti-CD66 (BD Biosciences, 1:500) and mouse anti-CD71 (H68.4 [Life Technologies], 1:1,000). For Western blotting, the following antibodies were used: mouse anti-PTEN (6H2.1 [Millipore], 1:5,000), mouse anti-β-catenin (BD Biosciences, 1:5,000), mouse anti-glyceraldehyde-3-phosphate dehydrogenase (anti-GAPDH) (6C5 [Millipore], 1:5,000) mouse anti-α-tubulin (Calbiochem, 1:5,000), mouse anti-Flag (M2 [Sigma], 1:10,000), mouse antihemagglutinin (anti-HA) (12CA5 [Roche], 1:10,000), mouse anti-GFP (clones 7.1 and 13.1 [Roche], 1:5,000), mouse anti-V5 (Invitrogen, 1:5,000), and rabbit anti-Fap1 (H-300 [Santa Cruz], 1:2,000).

### Generation of PTEN knockout and PTPL1 knockdown cell lines.

PTEN knockout Ls174T:W4 cells were generated using CRISPR/Cas9-mediated gene disruption. For this, cells were transfected with pSpCas9(BB)-2A-GFP (PX458), encoding a single guide RNA (sgRNA) (ACCGCCAAATTTAATTGCAG for clones 1 and 2 or GACTGGGAATAGTTACTCCC for clone 3) targeting the fourth and sixth exons of PTEN. GFP-positive cells were expanded monoclonally, and knockout clones were identified by sequencing. The absence of PTEN was subsequently demonstrated by Western blotting.

For stable knockdown of PTPL1, cells were transduced with short hairpin RNA (shRNA) containing lentiviral particles (Mission shRNA library Sigma). After two rounds of infection, cells were selected for puromycin resistance (10 μg/ml) and knockdown was confirmed by Western blotting and quantitative PCR (qPCR). For phenotypic analysis of PTPL1 knockdown, we used the pooled and three individual short hairpins with the following target sequence: shPTPL1 1, 5′-GCCACGGTCTATTCTTACTAA-3′; shPTPL1 2, 5′-GCATCATCTGTTTGTAATCAT-3′; and shPTPL1 3, 5′-CCAGAGTTTGAGGACAGTAAT-3′. For PTPL1 knockdown in the rescue experiments, a single shRNA hairpin (shPTPL1 2) targeting the 3′ untranslated region (3′UTR) of PTPL1 was used: 5′-GCATCATCTGTTTGTAATCAT-3′.

### qPCR.

RNA was isolated using the RNeasy minikit (Qiagen) according to the manufacturer's protocol; 1.5 μg of RNA was used for cDNA synthesis with the iScript cDNA synthesis kit (Bio-Rad), and this was subsequently used for qPCR with the FastStart universal SYBR green master mix (Roche). cDNA was amplified on a C1000 thermal cycler (Bio-Rad) with the following primers: PTPN13_fw (5′-CAAAGGTGATCGCGTCCTA-3′) and PTPN13_rv (5′-CGGGACATGTTCTTTAGATGTT-3′). Expression levels were normalized to GAPDH and HPRT1 mRNA levels. The data presented are the averages from two biological replicates which each contained three technical replicates.

### Live-cell imaging and image analysis.

Transfected cells were split and seeded onto glass-bottom dishes (WillCo Wells) in doxycycline-containing medium. Cells were imaged in HEPES-buffered (pH 7.4) Leibovitz's L-15 medium (Invitrogen) at 37°C using an Axioskop2 LSM510 scanning confocal microscope (Zeiss) with a ×63 magnification oil objective (Plan Apochromat; numerical aperture [NA], 1.4) using Zen image acquisition software. The apical membrane size was determined by measuring the fraction of the cell covered with microvilli using ImageJ software. Average apical membrane sizes were compared using independent sample *t* tests in SPSS with a *P* value of <0.05 as a cutoff for significance. The apical membrane size in organoids was determined as described previously (27).

For visualization of PIP gradients, images were analyzed using ImageJ software. After background subtraction, the cytosolic area was masked, and a ratio image was calculated by dividing the pixel intensity of either PH-PLCδ-RFP with GFP-Kras(CAAX) or PH-Akt-GFP with RFP-Kras(CAAX). This image was displayed in a false-coloring scheme using the “fire” lookup table.

Quantification of apical enrichment of YFP-PTEN was performed by making a line scan through the apical and basal membranes and determining the ratio between average apical and basal membrane pixel intensities using ImageJ. Average enrichment ratios were compared using independent sample *t* tests with a *P* value of <0.05 as a cutoff for significance.

### Immunofluorescence.

Cells were seeded on glass coverslips in the presence of doxycycline. Cells were washed with phosphate-buffered saline (PBS), fixed in 4% formaldehyde for 30 min, permeabilized with 0.1% Triton X-100 for 10 min, and blocked in 2% bovine serum albumin (BSA) for 2 h. Slides then incubated with primary antibody for 16 h, washed with PBS, and incubated with Alexa Fluor 488-conjugated secondary antibody in the presence of DAPI (4′,6′-diamidino-2-phenylindole) and Alexa Fluor 568-coupled phalloidin for at least 4 h. After multiple PBS washes, slides were mounted and imaged.

Staining of fixed organoids was performed as previously described (27). In brief, organoids were fixed in 4% formaldehyde for 15 min at 4°C, washed with PBS, and subsequently permeabilized in PBD-0.2T buffer (1% BSA, 10% dimethyl sulfoxide [DMSO], and 0.2% Triton X-100 in PBS). Organoids were incubated in PBD-0.2T buffer containing phalloidin-Alexa Fluor 568 and DAPI for at least 4 h, washed (2 times in PBD-0.2T and 2 times in PBS), and imaged.

### Immunoprecipitation.

Transfected HEK293T cells were scraped in ice-cold lysis buffer (1% Triton X-100, 50 mM Tris-HCl [pH 7.5], 150 mM NaCl, and 5 mM MgCl_2_, supplemented with protease inhibitors) and cleared by centrifugation. Cleared lysates were incubated with agarose beads coupled to GFP binding protein (GBP) for 1 h at 4°C while rotating. Beads were washed three times with lysis buffer, and bound proteins were eluted in sample buffer.
